# On the Motion of Substance in a Channel of a Network: Extended Model and New Classes of Probability Distributions

**DOI:** 10.3390/e22111240

**Published:** 2020-10-31

**Authors:** Nikolay K. Vitanov, Kaloyan N. Vitanov, Holger Kantz

**Affiliations:** 1 Institute of Mechanics, Bulgarian Academy of Sciences, Acad. G. Bonchev Str., Block 4, 1113 Sofia, Bulgaria; kalovitanov@gmail.com; 2 Max-Planck Institute for the Physics of Complex Systems, Noethnitzerstr. 38, 01187 Dresden, Germany; kantz@pks.mpg.de

**Keywords:** network, channel of network, flow of substance in a channel of a network, probability distributions for stationary flow of substance, Katz, Ord, Kemp families of probability distributions, general forms of discrete and continuous distribution for stationary flow of substance

## Abstract

We discuss the motion of substance in a channel containing nodes of a network. Each node of the channel can exchange substance with: (i) neighboring nodes of the channel, (ii) network nodes which do not belong to the channel, and (iii) environment of the network. The new point in this study is that we assume possibility for exchange of substance among flows of substance between nodes of the channel and: (i) nodes that belong to the network but do not belong to the channel and (ii) environment of the network. This leads to an extension of the model of motion of substance and the extended model contains previous models as particular cases. We use a discrete-time model of motion of substance and consider a stationary regime of motion of substance in a channel containing a finite number of nodes. As results of the study, we obtain a class of probability distributions connected to the amount of substance in nodes of the channel. We prove that the obtained class of distributions contains all truncated discrete probability distributions of discrete random variable ω which can take values 0,1,⋯,N. Theory for the case of a channel containing infinite number of nodes is presented in Appendix A. The continuous version of the discussed discrete probability distributions is described in Appendix B. The discussed extended model and obtained results can be used for the study of phenomena that can be modeled by flows in networks: motion of resources, traffic flows, motion of migrants, etc.

## 1. Introduction

Networks of different degrees of complexity arise often in research on complex systems. Flows of substances occur frequently in these systems and because of this, network flow models are used, for example, to study flows in computer networks [1], flows in electrical and communication networks [2], to detect network structure [3], to study flows in financial networks [4], flows connected to transportation problems [5,6,7], etc. [8,9,10,11,12]. Below, we discuss flow of substance in a channel of a network containing a chain of network’s nodes which are connected by edges. The kind of substance can be different (e.g., some resource which flows through the channel) and we consider a discrete-time model of motion of the substance through the studied channel. There are exchanges between nodes of the channel and (i) other nodes of the channel; (ii) network nodes which are not part of the channel; and (iii) environment of the network. The new point in this study is that in addition to the above exchanges we assume also possibility for exchange of substance among the flows between the nodes of the channel and (i) the network; (ii) the environment of the network. Thus, the discussed model extends models studied up to now, for example, in [13,14], and has different possible applications such as: (i) to model flow of a substance through a channel and use of part of this substance in some industrial process happening in the nodes of the channel or (ii) to model human migration flows. The last application is important because of the frequent use of probability and deterministic models of human migration [15,16,17,18,19] and as the migration flows are important for taking decisions about economic development of regions of a country [20,21,22,23,24] for analysis of migration networks [13,25,26,27,28,29,30,31], and ideological struggles [32,33]; for study of waves and probability distributions in population systems [34,35,36,37], etc. Migration-like models are also used in other research areas [38,39]. The model described below can be connected to an appropriate urn model. This is a useful connection as urn models are greatly applied in the research of various problems, e.g., genetics, clinical trials, biology, social dynamics, military theory, etc. [40,41,42,43].

The text below is organized as follows. In Section 2, we formulate a general model of motion of a substance in a channel of a network containing a finite number of nodes. In Section 3, we describe the particular case of the general model which will be discussed in the article. in Section 4 we obtain a class of probability distributions connected to the stationary motion of substance through the channel and show that this class of distributions contains all possible truncated discrete distributions of a random variable which can take values 0,⋯,N. As a result of this, the obtained class of distributions contains, in particular, cases of the classes of (long-tail) distributions obtained in our previous research. Short discussion of the obtained results is presented in Section 5. Theory for the case of the channel containing infinitely many nodes is presented in Appendix A. The class of probability distributions obtained there contains, in particular, cases of the classes of distributions of Katz, Ord, Kemp, etc. In Appendix B, we obtain a probability distribution for the case of a continuous random variable taking values between 0 and 1. This distribution is a continuous analogue of the discrete distributions discussed in main text and in Appendix A.

## 2. Mathematical Formulation of the Model

**Figure 1:**
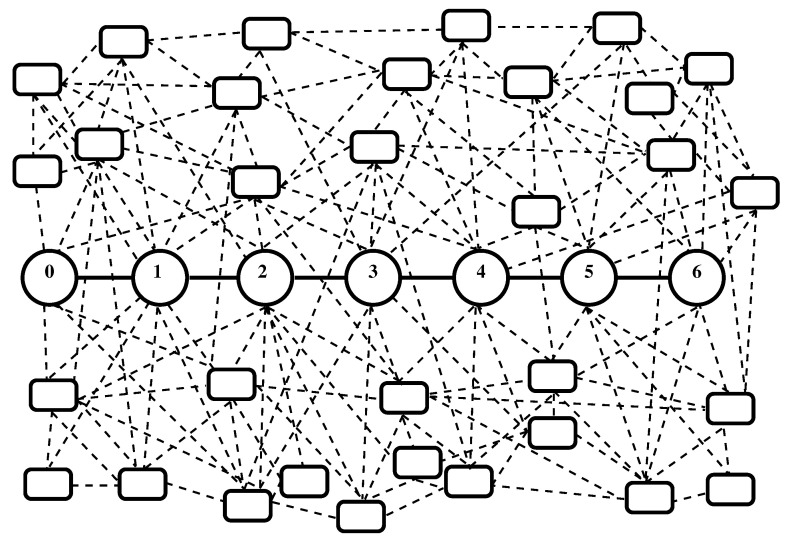
An example of the kind of channel studied in the text. The channel consists of 7 nodes labeled from 0 to 6. Some substance can move through the channel. Exchange of substance is possible between nodes of the channel, other nodes of the network, and environment of the network. In addition, there can be exchange of substance among the flows between the nodes of the channel and other nodes of the network or environment of the network. The nodes of the network that belong to the channel and edges that connect these nodes are painted by circles and solid lines. Other nodes and edges of the network are painted by rectangles and dashed lines.

We consider a channel in a network consisting of nodes connected by edges—Figure 1. We assume that the channel consists of a chain of N+1 nodes (labeled from 0 to *N*) connected by corresponding edges (ways for motion of the substance). Each edge connects two nodes and each node is connected to two edges except for 0-th node and *N*-th node which are connected to one edge. Some kind of substance moves through the nodes and edges of the channel.

**Figure 2:**
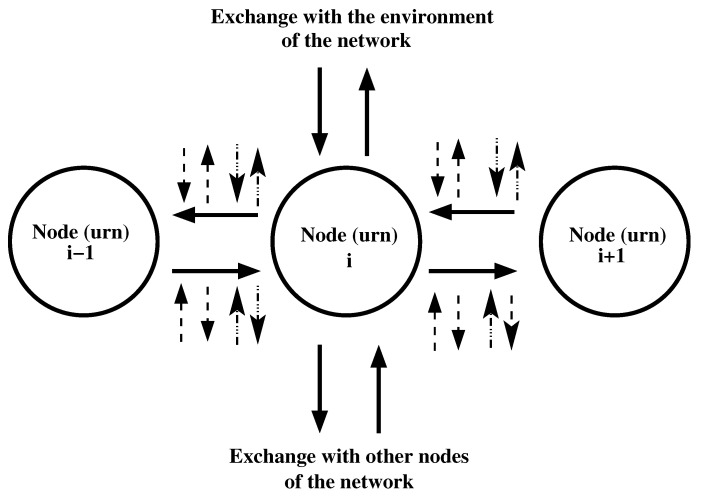
Exchanges connected with the *i*-th node of chain of nodes i=0,⋯,N. Note that nodes with numbers 0 and *N* are connected only to one of other nodes of the channel. These two nodes may exchange substance only with one of the other nodes of the channel. The new point in this study is the possibility for an exchange of substance: (i) among flows between nodes and the network (arrows with dashed lines) and (ii) among flows between nodes and environment of the network (arrows with dot-dashed lines).

This motion is accompanied by processes of the exchange of substance between the channel, the rest of the network, and the environment of the network. The possible processes of exchange of substance for the *i*-th node of the channel are shown in Figure 2. One can image that an urn is placed in any of the nodes of the channel and in any of other nodes of the network and exchange of substance is among these urns. The *i*-th node can exchange substance with the (i−1)-th and with (i+1)-th nodes. The *i*-th node can also exchange substance with network nodes outside the channel and with the environment of the network. Let us denote as: (i) “leakage”—the process of motion of substance from a node of the channel to a node of the network or from a node of the channel to environment of the network; (ii) “inflow”—the process of motion of substance from a node of the network that does not belong to the channel or from the environment of the network to a node of the channel. The additional exchanges of substance connected to the flows between the *i*-th node and other nodes are shown in Figure 2 by arrows having dashed or dot-dashed lines. These exchanges are not accounted for by the previous models.

We consider a discrete-time and assume that intervals between moments of time have equal length. At each time interval, the substance in a node of the channel can participate in the following processes:

(1) substance remains in the same node;
(2) substance moves to previous or to the next node (e.g., substance may move from node *m* to node m+1 or from node *m* to node m−1);
(3) substance leaks from the node *m*. Leaked substance does not belong anymore to the channel. Such substance may spread through the network or through the environment of the network. Thus, we have two kinds of leakage: (i) leakage from node *m* of the channel to nodes of the network and (ii) leakage from node *m* of the channel to the environment of the network;
(4) the substance “inflows” to node *m*. Two kinds of “inflow” are possible: (i) inflow from nodes of the network to the node *m* of the channel and (ii) inflow from environment of the network to the node *m* of the channel.

In addition, the substance from any of the flows between nodes of the channel can participate in the following processes:

(5) substance can move from the flow to a node of the network which does not belong to the channel (arrows with dashed line pointing from the flow in Figure 2);
(6) substance can move from a node of the network which does not belong to the channel to the flow between two nodes of the channel (arrows with dashed line pointing to the flow in Figure 2);
(7) substance can move from the flow to the environment of the network (arrows with dot-dashed line pointing from the flow in Figure 2);
(8) substance can move from the environment of the network to the flow between two nodes of the channel ( arrows with dot-dashed line pointing to the flow in Figure 2).

Let us formalize mathematically the above considerations. We consider discrete-time $t_{k}$, k=0,1,2,⋯ and denote by $x_{i}\left(t_{k}\right)$ the amount of substance in the *i*-th node of the channel at the beginning of time interval $\left[t_{k},t_{k}+\Delta t\right]$. For processes happening in this time interval in the *n*-th node of the channel, we use the following notations:

1. $i_{n}^{e}\left(t_{k}\right)$ and $o_{n}^{e}\left(t_{k}\right)$ are amounts of inflow and outflow of substance from the environment to the *n*-th node of the channel (upper index *e* means that the quantities are for the environment);
2. $o_{n}^{c}\left(t_{k}\right)$ is the amount of outflow of substance from the *n*-th node of the channel to the (n+1)-th node of the channel (upper index *c* means that the quantity is for the channel);
3. $i_{n}^{c}\left(t_{k}\right)$ is the amount of inflow of substance from the (n+1) node of the channel to the *n*-th node of the channel;
4. $o_{n}^{n}\left(t_{k}\right)$ and $i_{n}^{n}\left(t_{k}\right)$ are the amounts of outflow and inflow of substance between the *n*-th node of the channel and the network (upper index *n* means that quantities are for the network).

In addition, there are the following exchanges for the flows between nodes of the channel, nodes of the network, and environment of the network. We note that in general we have two flows of substance between the nodes of the channel: (a) flow directed from *i*-th node of the channel to i+1-th node of the channel, and (b) flow directed from i+1-th node of the channel to *i*-th node of the channel. We denote the corresponding flows as follows:

5. $f_{n}^{e}\left(t_{k}\right)$: amount of the outflow of substance to the environment of network from the flow between the *n*-th and n+1-th nodes of the channel;
6. $f_{n}^{n}\left(t_{k}\right)$: amount of the outflow of substance to the network from the flow between the *n*-th and n+1-th nodes of the channel;
7. $g_{n}^{e}\left(t_{k}\right)$: amount of the inflow of substance from the environment of the network to the flow between the *n*-th and n+1-th nodes of the channel;
8. $g_{n}^{n}\left(t_{k}\right)$: amount of the inflow of substance from the network to the flow between the *n*-th and n+1-th nodes of the channel;
9. $h_{n}^{e}\left(t_{k}\right)$: amount of the outflow of substance to the environment of network from the flow between the n+1-th and *n*-th nodes of the channel;
10. $h_{n}^{n}\left(t_{k}\right)$: amount of the outflow of substance to the network from the flow between the n+1-th and *n*-th node of the channel;
11. $p_{n}^{e}\left(t_{k}\right)$: amount of the inflow of substance from the environment of the network to the flow between the n+1-th and *n*-th nodes of the channel;
12. $p_{n}^{n}\left(t_{k}\right)$: amount of the inflow of substance from the network to the flow between the n+1-th and *n*-th nodes of the channel.

We are interested in the amounts of substance available in the nodes of the channel. For the 0-th node there are: exchange of substance with the environment (inflow and outflow); exchange of substance with next node of channel (inflow and outflow); and exchange of substance with the network (inflow and outflow). In addition to the inflow from the neighbor node of the channel, there are exchanges of substance (inflow and outflow) with the network and with the environment of the network. Similarly, 4 exchanges are available also for the outflow from the 0-th node of the channel to the next node of the channel. From all of these exchanges, what are significant for the 0-th node are the inflows and outflows from the network and from the environment of the network towards the inflow of substance in the 0-th node as these exchanges of substance contribute to the change of substance in the 0-th node of the channel. Thus the change of amount of substance in 0-th node of channel is described by the relationship

(1) $$\begin{array}{ccc} x_{0}\left(t_{k+1}\right) & = & x_{0}\left(t_{k}\right)+i_{0}^{e}\left(t_{k}\right)-o_{0}^{e}\left(t_{k}\right)-o_{0}^{c}\left(t_{k}\right)+i_{0}^{c}\left(t_{k}\right)-o_{0}^{n}\left(t_{k}\right)+i_{0}^{n}\left(t_{k}\right)-h_{0}^{e}\left(t_{k}\right)-\\ & & h_{0}^{n}\left(t_{k}\right)+p_{0}^{e}\left(t_{k}\right)+p_{0}^{n}\left(t_{k}\right). \end{array}$$

For the nodes of the channel numbered by i=1,⋯,N−1, there is an exchange of substance with the environment, exchange of substance with the network, and exchange of substance with the (i−1)-th and (i+1)-th node of the channel. Thus, the change of amount of substance in the *i*-th node of channel is described by relationship

(2) $$\begin{array}{ccc} x_{i}\left(t_{k+1}\right) & = & x_{i}\left(t_{k}\right)+i_{i}^{e}\left(t_{k}\right)-o_{i}^{e}\left(t_{k}\right)+o_{i-1}^{c}\left(t_{k}\right)-i_{i-1}^{c}\left(t_{k}\right)-o_{i}^{c}\left(t_{k}\right)+i_{i}^{c}\left(t_{k}\right)-o_{i}^{n}\left(t_{k}\right)+i_{i}^{n}\left(t_{k}\right)-\\ & & h_{i}^{e}\left(t_{k}\right)-h_{i}^{n}\left(t_{k}\right)+p_{i}^{e}\left(t_{k}\right)+p_{i}^{n}\left(t_{k}\right)-f_{i-1}^{e}\left(t_{k}\right)-f_{i-1}^{n}\left(t_{k}\right)+g_{i-1}^{e}\left(t_{k}\right)+g_{i-1}^{n}\left(t_{k}\right),\\ & & i=1,\cdots ,N-1 \end{array}$$

For the *N*-th node of the channel, there is an exchange of substance with the environment, exchange of substance with the network, and exchange of substance with the (N−1)-th node of the channel. Thus, the change of the amount of substance in the *N*-th node of channel is described by relationship

(3) $$\begin{array}{ccc} x_{N}\left(t_{k+1}\right) & = & x_{N}\left(t_{k}\right)+i_{N}^{e}\left(t_{k}\right)-o_{N}^{e}\left(t_{k}\right)+o_{N-1}^{c}\left(t_{k}\right)-i_{N-1}^{c}\left(t_{k}\right)-o_{N}^{n}\left(t_{k}\right)+i_{N}^{n}\left(t_{k}\right)-f_{N-1}^{e}\left(t_{k}\right)-\\ & & f_{N-1}^{n}\left(t_{k}\right)+g_{N-1}^{e}\left(t_{k}\right)+g_{N-1}^{n}\left(t_{k}\right). \end{array}$$

Equations (1)–(3) describe the general case of motion of substance along a channel of nodes connected to a network and in direction to the environment of this network.

## 3. Studied Particular Case of the General Model (1)–(3)

We continue our study by consideration of a particular case for the relationships for the quantities from the system of Equations (1)–(3). In this particular case, we have linear relationships connecting the exchanges between the nodes of the channel and the amounts of substance in the nodes. The relationships are as follows.

- Exchange between the nodes of the channel and the environment of the network
  (4) $$\begin{array}{ccc} i_{0}^{e}\left(t_{k}\right) & = & \sigma_{0}\left(t_{k}\right)x_{0}\left(t_{k}\right);\;\;o_{0}^{e}\left(t_{k}\right)=\mu_{0}\left(t_{k}\right)x_{0}\left(t_{k}\right)\\ i_{i}^{e}\left(t_{k}\right) & = & \sigma_{i}\left(t_{k}\right)x_{i}\left(t_{k}\right);\;\;o_{i}^{e}\left(t_{k}\right)=\mu_{i}\left(t_{k}\right)x_{i}\left(t_{k}\right),\;\;i=1,\cdots ,N-1\\ i_{N}^{e}\left(t_{k}\right) & = & \sigma_{N}\left(t_{k}\right)x_{N}\left(t_{k}\right);\;\;o_{N}^{e}\left(t_{k}\right)=\mu_{N}\left(t_{k}\right)x_{N}\left(t_{k}\right) \end{array}$$
- Exchange between the channel and the network
  (5) $$\begin{array}{ccc} i_{0}^{n}\left(t_{k}\right) & = & \epsilon_{0}\left(t_{k}\right)x_{0}\left(t_{k}\right);\;\;o_{0}^{n}\left(t_{k}\right)=\gamma_{0}\left(t_{k}\right)x_{0}\left(t_{k}\right)\\ i_{i}^{n}\left(t_{k}\right) & = & \epsilon_{i}\left(t_{k}\right)x_{i}\left(t_{k}\right);\;\;o_{i}^{n}\left(t_{k}\right)=\gamma_{i}\left(t_{k}\right)x_{i}\left(t_{k}\right),\;\;i=1,\cdots ,N-1\\ i_{N}^{n}\left(t_{k}\right) & = & \epsilon_{N}\left(t_{k}\right)x_{N}\left(t_{k}\right);\;\;o_{N}^{n}\left(t_{k}\right)=\gamma_{N}\left(t_{k}\right)x_{N}\left(t_{k}\right) \end{array}$$
- Exchange within the channel
  (6) $$\begin{array}{ccc} o_{0}^{c}\left(t_{k}\right) & = & \varphi_{0}\left(t_{k}\right)x_{0}\left(t_{k}\right);\;i_{0}^{c}\left(t_{k}\right)=\delta_{1}\left(t_{k}\right)x_{1}\left(t_{k}\right)\\ o_{i}^{c}\left(t_{k}\right) & = & \varphi_{i}\left(t_{k}\right)x_{i}\left(t_{k}\right);\;i_{i}^{c}\left(t_{k}\right)=\delta_{i+1}\left(t_{k}\right)x_{i+1}\left(t_{k}\right),\;\;i=1,\cdots ,N-2\\ o_{N-1}^{c}\left(t_{k}\right) & = & \varphi_{N-1}\left(t_{k}\right)x_{N-1}\left(t_{k}\right);\\ i_{N-1}^{c}\left(t_{k}\right) & = & \delta_{N}\left(t_{k}\right)x_{N}\left(t_{k}\right) \end{array}$$
- Exchanges between flows among the nodes of the channel and the network/environment of the network
  (7) $$\begin{array}{ccc} f_{n}^{e}\left(t_{k}\right) & = & \zeta_{n}\left(t_{k}\right);\;\;f_{n}^{n}\left(t_{k}\right)=\theta_{n}\left(t_{k}\right);\\ g_{n}^{e}\left(t_{k}\right) & = & \kappa_{n}\left(t_{k}\right);\;\;g_{n}^{n}\left(t_{k}\right)=\lambda_{n}\left(t_{k}\right)\\ h_{n}^{e}\left(t_{k}\right) & = & \nu_{n}\left(t_{k}\right);\;\;h_{n}^{n}\left(t_{k}\right)=\pi_{n}\left(t_{k}\right)\\ p_{n}^{e}\left(t_{k}\right) & = & \rho_{n}\left(t_{k}\right);\;\;p_{n}^{n}\left(t_{k}\right)=\tau_{n}\left(t_{k}\right) \end{array}$$

For this particular case, the system of Equations (1)–(3) becomes

(8) $$\begin{array}{ccc} x_{0}\left(t_{k+1}\right) & = & x_{0}\left(t_{k}\right)+\sigma_{0}\left(t_{k}\right)x_{0}\left(t_{k}\right)-\mu_{0}\left(t_{k}\right)x_{0}\left(t_{k}\right)-\varphi_{0}\left(t_{k}\right)x_{0}\left(t_{k}\right)+\delta_{1}\left(t_{k}\right)x_{1}\left(t_{k}\right)-\\ & & \gamma_{0}\left(t_{k}\right)x_{0}\left(t_{k}\right)+\epsilon_{0}\left(t_{k}\right)x_{0}\left(t_{k}\right)-\nu_{0}\left(t_{k}\right)-\pi_{0}\left(t_{k}\right)+\rho_{0}\left(t_{k}\right)+\tau_{0}\left(t_{k}\right), \end{array}$$

(9) $$\begin{array}{ccc} x_{i}\left(t_{k+1}\right) & = & x_{i}\left(t_{k}\right)+\sigma_{i}\left(t_{k}\right)x_{i}\left(t_{k}\right)-\mu_{i}\left(t_{k}\right)x_{i}\left(t_{k}\right)+\varphi_{i-1}\left(t_{k}\right)x_{i-1}\left(t_{k}\right)-\delta_{i}\left(t_{k}\right)x_{i}\left(t_{k}\right)-\\ & & \varphi_{i}\left(t_{k}\right)x_{i}\left(t_{k}\right)+\delta_{i+1}\left(t_{k}\right)x_{i+1}\left(t_{k}\right)-\gamma_{i}\left(t_{k}\right)x_{i}\left(t_{k}\right)+\epsilon_{i}\left(t_{k}\right)x_{i}\left(t_{k}\right)-\nu_{i}\left(t_{k}\right)-\\ & & \pi_{i}\left(t_{k}\right)+\rho_{i}\left(t_{k}\right)+\tau_{i}\left(t_{k}\right)-\zeta_{i-1}\left(t_{k}\right)-\theta_{i-1}\left(t_{k}\right)+\kappa_{i-1}\left(t_{k}\right)+\lambda_{i-1}\left(t_{k}\right),\\ & & i=1,\cdots ,N-1, \end{array}$$

$$\begin{array}{ccc} x_{N}\left(t_{k+1}\right) & = & x_{N}\left(t_{k}\right)+\sigma_{N}\left(t_{k}\right)x_{N}\left(t_{k}\right)-\mu_{N}\left(t_{k}\right)x_{i}\left(t_{k}\right)+\varphi_{N-1}\left(t_{k}\right)x_{N-1}\left(t_{k}\right)-\delta_{N}\left(t_{k}\right)x_{N}\left(t_{k}\right)-\\ & & \gamma_{N}\left(t_{k}\right)x_{N}\left(t_{k}\right)+\epsilon_{N}\left(t_{k}\right)x_{N}\left(t_{k}\right)-\zeta_{N-1}\left(t_{k}\right)-\theta_{N-1}\left(t_{k}\right)+\kappa_{N-1}\left(t_{k}\right)+\lambda_{N-1}\left(t_{k}\right). \end{array}$$

Below, we consider the case of absence of an inflow from the (i+1)-th node to the *i*-th node of the channel (no flow of substance in the direction opposite to the direction from the 0-th node to the *N*-th node of the channel). In this case, the system of Equations (8)–(10) becomes

(10) $$\begin{array}{ccc} x_{0}\left(t_{k+1}\right) & = & x_{0}\left(t_{k}\right)+\sigma_{0}\left(t_{k}\right)x_{0}\left(t_{k}\right)-\mu_{0}\left(t_{k}\right)x_{0}\left(t_{k}\right)-\varphi_{0}\left(t_{k}\right)x_{0}\left(t_{k}\right)-\gamma_{0}\left(t_{k}\right)x_{0}\left(t_{k}\right)\\ & & +\epsilon_{0}\left(t_{k}\right)x_{0}\left(t_{k}\right) \end{array}$$

(11) $$\begin{array}{ccc} x_{i}\left(t_{k+1}\right) & = & x_{i}\left(t_{k}\right)+\sigma_{i}\left(t_{k}\right)x_{i}\left(t_{k}\right)-\mu_{i}\left(t_{k}\right)x_{i}\left(t_{k}\right)+\varphi_{i-1}\left(t_{k}\right)x_{i-1}\left(t_{k}\right)-\varphi_{i}\left(t_{k}\right)x_{i}\left(t_{k}\right)-\\ & & \gamma_{i}\left(t_{k}\right)x_{i}\left(t_{k}\right)+\epsilon_{i}\left(t_{k}\right)x_{i}\left(t_{k}\right)-\zeta_{i-1}\left(t_{k}\right)-\theta_{i-1}\left(t_{k}\right)+\kappa_{i-1}\left(t_{k}\right)+\lambda_{i-1}\left(t_{k}\right)\\ & & i=1,\cdots ,N-1 \end{array}$$

(12) $$\begin{array}{ccc} x_{N}\left(t_{k+1}\right) & = & x_{N}\left(t_{k}\right)+\sigma_{N}\left(t_{k}\right)x_{N}\left(t_{k}\right)-\mu_{N}\left(t_{k}\right)x_{i}\left(t_{k}\right)+\varphi_{N-1}\left(t_{k}\right)x_{N-1}\left(t_{k}\right)-\gamma_{N}\left(t_{k}\right)x_{N}\left(t_{k}\right)+\\ & & \epsilon_{N}\left(t_{k}\right)x_{N}\left(t_{k}\right)-\zeta_{N-1}\left(t_{k}\right)-\theta_{N-1}\left(t_{k}\right)+\kappa_{N-1}\left(t_{k}\right)+\lambda_{N-1}\left(t_{k}\right) \end{array}$$

We shall study the stationary case of the model Equations (10)–(12) in more detail below.

## 4. Results

We consider the case of stationary motion of the substance through the channel of nodes. In this case, $x_{i}\left(t_{k+1}\right)=x_{i}\left(t_{k}\right)$, i=0,⋯,N and the system of Equations (10)–(12) becomes

(13) $$\begin{array}{c} \left[\sigma_{0}\left(t_{k}\right)-\mu_{0}\left(t_{k}\right)-\varphi_{0}\left(t_{k}\right)-\gamma_{0}\left(t_{k}\right)+\epsilon_{0}\left(t_{k}\right)\right]x_{0}\left(t_{k}\right)=0 \end{array}$$

(14) $$\begin{array}{c} \left[\mu_{i}\left(t_{k}\right)+\varphi_{i}\left(t_{k}\right)+\gamma_{i}\left(t_{k}\right)-\sigma_{i}\left(t_{k}\right)-\epsilon_{i}\left(t_{k}\right)\right]x_{i}\left(t_{k}\right)=\varphi_{i-1}\left(t_{k}\right)x_{i-1}\left(t_{k}\right)+(-\zeta_{i-1}\left(t_{k}\right)-\theta_{i-1}\left(t_{k}\right)+\\ \kappa_{i-1}\left(t_{k}\right)+\lambda_{i-1}\left(t_{k}\right)),\;i=1,\cdots ,N-1 \end{array}$$

(15) $$\begin{array}{c} \left[\mu_{N}\left(t_{k}\right)+\gamma_{N}\left(t_{k}\right)-\sigma_{N}\left(t_{k}\right)-\epsilon_{N}\left(t_{k}\right)\right]x_{N}\left(t_{k}\right)=\varphi_{N-1}\left(t_{k}\right)x_{N-1}\left(t_{k}\right)+(-\zeta_{N-1}\left(t_{k}\right)-\theta_{N-1}\left(t_{k}\right)+\\ \kappa_{N-1}\left(t_{k}\right)+\lambda_{N-1}\left(t_{k}\right)) \end{array}$$

Below we discuss the model described by Equations (13)–(15) for the case when the parameters of the model are time independent (i.e., when $\sigma_{i}\left(t_{k}\right)=\sigma$; $\mu_{i}\left(t_{k}\right)=\mu_{i}$; $\gamma_{i}\left(t_{k}\right)=\gamma_{i}$; $\epsilon_{i}\left(t_{k}\right)=\epsilon_{i}$; $f_{0}\left(t_{k}\right)=f_{0}$; i=0,⋯,N. Note that these parameters do not depend on time but they may depend on *i* and also on other parameters connected to the network and to the environment of the network. In this case, the system of equations becomes

(16) $$\begin{array}{c} \left[\sigma_{0}-\mu_{0}-\varphi_{0}-\gamma_{0}+\epsilon_{0}\right]x_{0}=0 \end{array}$$

(17) $$\begin{array}{c} \left[\mu_{i}+\varphi_{i}+\gamma_{i}-\sigma_{i}-\epsilon_{i}\right]x_{i}=\varphi_{i-1}x_{i-1}+\left(-\zeta_{i-1}-\theta_{i-1}+\kappa_{i-1}+\lambda_{i-1}\right),i=1,\cdots ,N-1 \end{array}$$

(18) $$\begin{array}{c} \left[\mu_{N}+\gamma_{N}-\sigma_{N}-\epsilon_{N}\right]x_{N}=\varphi_{N-1}x_{N-1}+\left(-\zeta_{N-1}-\theta_{N-1}+\kappa_{N-1}+\lambda_{N-1}\right) \end{array}$$

From the system of Equations (16)–(18), we obtain the following relationships (note that $\delta_{i,j}$ below is the Kronecker delta symbol):

$$\begin{array}{ccc} \varphi_{0} & = & \sigma_{0}-\mu_{0}-\gamma_{0}+\epsilon_{0}, \end{array}$$

$$\begin{array}{ccc} x_{k} & = & x_{0}\prod_{i=1}^{k}\frac{\varphi_{i-1}}{\mu_{i}+\varphi_{i}+\gamma_{i}-\sigma_{i}-\epsilon_{i}}+\\ & & \left(1-\delta_{k1}\right)\sum_{j=1}^{k-1}\left(\frac{\kappa_{j-1}+\lambda_{j-1}-\zeta_{j-1}-\theta_{j-1}}{\mu_{j}+\varphi_{j}+\gamma_{j}-\sigma_{j}-\epsilon_{j}}\prod_{l=j+1}^{k}\frac{\varphi_{l-1}}{\mu_{l}+\varphi_{l}+\gamma_{l}-\sigma_{l}-\epsilon_{l}}\right)+\\ & & \frac{\kappa_{k-1}+\lambda_{k-1}-\zeta_{k-1}-\theta_{k-1}}{\mu_{k}+\varphi_{k}+\gamma_{k}-\sigma_{k}-\epsilon_{k}},\;\;\;k=1,\cdots ,N-1, \end{array}$$

(19) $$\begin{array}{ccc} x_{N} & = & x_{0}\frac{\varphi_{N-1}}{\mu_{N}+\gamma_{N}-\sigma_{N}-\epsilon_{N}}\prod_{i=1}^{N-1}\frac{\varphi_{i-1}}{\mu_{i}+\varphi_{i}+\gamma_{i}-\sigma_{i}-\epsilon_{i}}+\\ & & \frac{\varphi_{N-1}}{\mu_{N}+\gamma_{N}-\sigma_{N}-\epsilon_{N}}\left(1-\delta_{N,2}\right)\sum_{j=1}^{N-2}\left(\frac{\kappa_{j-1}+\lambda_{j-1}-\zeta_{j-1}-\theta_{j-1}}{\mu_{j}+\varphi_{j}+\gamma_{j}-\sigma_{j}-\epsilon_{j}}\prod_{l=j+1}^{N-1}\frac{\varphi_{l-1}}{\mu_{l}+\varphi_{l}+\gamma_{l}-\sigma_{l}-\epsilon_{l}}\right)+\\ & & \frac{\varphi_{N-1}}{\mu_{N}+\gamma_{N}-\sigma_{N}-\epsilon_{N}}\frac{\kappa_{N-2}+\lambda_{N-2}-\zeta_{N-2}-\theta_{N-2}}{\mu_{N-1}+\varphi_{N-1}+\gamma_{N-1}-\sigma_{N-1}-\epsilon_{N-1}}+\frac{\kappa_{N-1}+\lambda_{N-1}-\zeta_{N-1}-\theta_{N-1}}{\mu_{N}+\gamma_{N}-\sigma_{N}-\epsilon_{N}}, \end{array}$$

and

(20) $$\begin{array}{ccc} x & = & \sum_{k=0}^{N}x_{k}=x_{0}\left[1+\sum_{k=1}^{N-1}\prod_{i=1}^{k}\frac{\varphi_{i-1}}{\mu_{i}+\varphi_{i}+\gamma_{i}-\sigma_{i}-\epsilon_{i}}+\frac{\varphi_{N-1}}{\mu_{N}+\gamma_{N}-\sigma_{N}-\epsilon_{N}}\prod_{i=1}^{N-1}\frac{\varphi_{i-1}}{\mu_{i}+\varphi_{i}+\gamma_{i}-\sigma_{i}-\epsilon_{i}}\right]+\\ & & \sum_{k=1}^{N-1}[\left(1-\delta_{k,1}\right)\sum_{j=1}^{k-1}\left(\frac{\kappa_{j-1}+\lambda_{j-1}-\zeta_{j-1}-\theta_{j-1}}{\mu_{j}+\varphi_{j}+\gamma_{j}-\sigma_{j}-\epsilon_{j}}\prod_{l=j+1}^{k}\frac{\varphi_{l-1}}{\mu_{l}+\varphi_{l}+\gamma_{l}-\sigma_{l}-\epsilon_{l}}\right)+\\ & & \frac{\kappa_{k-1}+\lambda_{k-1}-\zeta_{k-1}-\theta_{k-1}}{\mu_{k}+\varphi_{k}+\gamma_{k}-\sigma_{k}-\epsilon_{k}}]+\frac{\varphi_{N-1}}{\mu_{N}+\gamma_{N}-\sigma_{N}-\epsilon_{N}}\left(1-\delta_{N,2}\right)\sum_{j=1}^{N-2}(\frac{\kappa_{j-1}+\lambda_{j-1}-\zeta_{j-1}-\theta_{j-1}}{\mu_{j}+\varphi_{j}+\gamma_{j}-\sigma_{j}-\epsilon_{j}}\times \\ & & \prod_{l=j+1}^{N-1}\frac{\varphi_{l-1}}{\mu_{l}+\varphi_{l}+\gamma_{l}-\sigma_{l}-\epsilon_{l}})+\frac{\varphi_{N-1}}{\mu_{N}+\gamma_{N}-\sigma_{N}-\epsilon_{N}}\frac{\kappa_{N-2}+\lambda_{N-2}-\zeta_{N-2}-\theta_{N-2}}{\mu_{N-1}+\varphi_{N-1}+\gamma_{N-1}-\sigma_{N-1}-\epsilon_{N-1}}+\\ & & \frac{\kappa_{N-1}+\lambda_{N-1}-\zeta_{N-1}-\theta_{N-1}}{\mu_{N}+\gamma_{N}-\sigma_{N}-\epsilon_{N}}. \end{array}$$

Equations (19) and (20) lead to a class of probability distributions as follows. We have $x_{i}$ and *x* and we can consider probability distribution $y_{i}=x_{i}/x$ connected to amount of substance in nodes of the channel. $y_{i}$ can be considered as probability values of distribution of a discrete random variable ω: $y_{i}=p\left(\omega =i\right)$, i=0,⋯,N. For this distribution, we obtain

(21) $$\begin{array}{ccc} y_{0} & = & x_{0}/\{x_{0}\left[1+\sum_{k=1}^{N-1}\prod_{i=1}^{k}\frac{\varphi_{i-1}}{\mu_{i}+\varphi_{i}+\gamma_{i}-\sigma_{i}-\epsilon_{i}}+\frac{\varphi_{N-1}}{\mu_{N}+\gamma_{N}-\sigma_{N}-\epsilon_{N}}\prod_{i=1}^{N-1}\frac{\varphi_{i-1}}{\mu_{i}+\varphi_{i}+\gamma_{i}-\sigma_{i}-\epsilon_{i}}\right]+\\ & & \sum_{k=1}^{N-1}[\left(1-\delta_{k,1}\right)\sum_{j=1}^{k-1}\left(\frac{\kappa_{j-1}+\lambda_{j-1}-\zeta_{j-1}-\theta_{j-1}}{\mu_{j}+\varphi_{j}+\gamma_{j}-\sigma_{j}-\epsilon_{j}}\prod_{l=j+1}^{k}\frac{\varphi_{l-1}}{\mu_{l}+\varphi_{l}+\gamma_{l}-\sigma_{l}-\epsilon_{l}}\right)+\\ & & \frac{\kappa_{k-1}+\lambda_{k-1}-\zeta_{k-1}-\theta_{k-1}}{\mu_{k}+\varphi_{k}+\gamma_{k}-\sigma_{k}-\epsilon_{k}}]+\frac{\varphi_{N-1}}{\mu_{N}+\gamma_{N}-\sigma_{N}-\epsilon_{N}}\left(1-\delta_{N,2}\right)\sum_{j=1}^{N-2}(\frac{\kappa_{j-1}+\lambda_{j-1}-\zeta_{j-1}-\theta_{j-1}}{\mu_{j}+\varphi_{j}+\gamma_{j}-\sigma_{j}-\epsilon_{j}}\times \\ & & \prod_{l=j+1}^{N-1}\frac{\varphi_{l-1}}{\mu_{l}+\varphi_{l}+\gamma_{l}-\sigma_{l}-\epsilon_{l}})+\frac{\varphi_{N-1}}{\mu_{N}+\gamma_{N}-\sigma_{N}-\epsilon_{N}}\frac{\kappa_{N-2}+\lambda_{N-2}-\zeta_{N-2}-\theta_{N-2}}{\mu_{N-1}+\varphi_{N-1}+\gamma_{N-1}-\sigma_{N-1}-\epsilon_{N-1}}+\\ & & \frac{\kappa_{N-1}+\lambda_{N-1}-\zeta_{N-1}-\theta_{N-1}}{\mu_{N}+\gamma_{N}-\sigma_{N}-\epsilon_{N}}\}, \end{array}$$

(22) $$\begin{array}{ccc} y_{k} & = & \{x_{0}\prod_{i=1}^{k}\frac{\varphi_{i-1}}{\mu_{i}+\varphi_{i}+\gamma_{i}-\sigma_{i}-\epsilon_{i}}+\left(1-\delta_{k1}\right)\sum_{j=1}^{k-1}\left(\frac{\kappa_{j-1}+\lambda_{j-1}-\zeta_{j-1}-\theta_{j-1}}{\mu_{j}+\varphi_{j}+\gamma_{j}-\sigma_{j}-\epsilon_{j}}\prod_{l=j+1}^{k}\frac{\varphi_{l-1}}{\mu_{l}+\varphi_{l}+\gamma_{l}-\sigma_{l}-\epsilon_{l}}\right)+\\ & & \frac{\kappa_{k-1}+\lambda_{k-1}-\zeta_{k-1}-\theta_{k-1}}{\mu_{k}+\varphi_{k}+\gamma_{k}-\sigma_{k}-\epsilon_{k}}\}/\{x_{0}[1+\sum_{k=1}^{N-1}\prod_{i=1}^{k}\frac{\varphi_{i-1}}{\mu_{i}+\varphi_{i}+\gamma_{i}-\sigma_{i}-\epsilon_{i}}+\\ & & \frac{\varphi_{N-1}}{\mu_{N}+\gamma_{N}-\sigma_{N}-\epsilon_{N}}\prod_{i=1}^{N-1}\frac{\varphi_{i-1}}{\mu_{i}+\varphi_{i}+\gamma_{i}-\sigma_{i}-\epsilon_{i}}]+\\ & & \sum_{k=1}^{N-1}\left[\left(1-\delta_{k,1}\right)\sum_{j=1}^{k-1}\left(\frac{\kappa_{j-1}+\lambda_{j-1}-\zeta_{j-1}-\theta_{j-1}}{\mu_{j}+\varphi_{j}+\gamma_{j}-\sigma_{j}-\epsilon_{j}}\prod_{l=j+1}^{k}\frac{\varphi_{l-1}}{\mu_{l}+\varphi_{l}+\gamma_{l}-\sigma_{l}-\epsilon_{l}}\right)+\frac{\kappa_{k-1}+\lambda_{k-1}-\zeta_{k-1}-\theta_{k-1}}{\mu_{k}+\varphi_{k}+\gamma_{k}-\sigma_{k}-\epsilon_{k}}\right]+\\ & & \frac{\varphi_{N-1}}{\mu_{N}+\gamma_{N}-\sigma_{N}-\epsilon_{N}}\left(1-\delta_{N,2}\right)\sum_{j=1}^{N-2}\left(\frac{\kappa_{j-1}+\lambda_{j-1}-\zeta_{j-1}-\theta_{j-1}}{\mu_{j}+\varphi_{j}+\gamma_{j}-\sigma_{j}-\epsilon_{j}}\prod_{l=j+1}^{N-1}\frac{\varphi_{l-1}}{\mu_{l}+\varphi_{l}+\gamma_{l}-\sigma_{l}-\epsilon_{l}}\right)+\\ & & \frac{\varphi_{N-1}}{\mu_{N}+\gamma_{N}-\sigma_{N}-\epsilon_{N}}\frac{\kappa_{N-2}+\lambda_{N-2}-\zeta_{N-2}-\theta_{N-2}}{\mu_{N-1}+\varphi_{N-1}+\gamma_{N-1}-\sigma_{N-1}-\epsilon_{N-1}}+\frac{\kappa_{N-1}+\lambda_{N-1}-\zeta_{N-1}-\theta_{N-1}}{\mu_{N}+\gamma_{N}-\sigma_{N}-\epsilon_{N}}\},\\ & & k=1,\cdots ,N-1, \end{array}$$

(23) $$\begin{array}{ccc} y_{N} & = & \{x_{0}\frac{\varphi_{N-1}}{\mu_{N}+\gamma_{N}-\sigma_{N}-\epsilon_{N}}\prod_{i=1}^{N-1}\frac{\varphi_{i-1}}{\mu_{i}+\varphi_{i}+\gamma_{i}-\sigma_{i}-\epsilon_{i}}+\\ & & \frac{\varphi_{N-1}}{\mu_{N}+\gamma_{N}-\sigma_{N}-\epsilon_{N}}\left(1-\delta_{N,2}\right)\sum_{j=1}^{N-2}\left(\frac{\kappa_{j-1}+\lambda_{j-1}-\zeta_{j-1}-\theta_{j-1}}{\mu_{j}+\varphi_{j}+\gamma_{j}-\sigma_{j}-\epsilon_{j}}\times \prod_{l=j+1}^{N-1}\frac{\varphi_{l-1}}{\mu_{l}+\varphi_{l}+\gamma_{l}-\sigma_{l}-\epsilon_{l}}\right)+\\ & & \frac{\varphi_{N-1}}{\mu_{N}+\gamma_{N}-\sigma_{N}-\epsilon_{N}}\frac{\kappa_{N-2}+\lambda_{N-2}-\zeta_{N-2}-\theta_{N-2}}{\mu_{N-1}+\varphi_{N-1}+\gamma_{N-1}-\sigma_{N-1}-\epsilon_{N-1}}+\frac{\kappa_{N-1}+\lambda_{N-1}-\zeta_{N-1}-\theta_{N-1}}{\mu_{N}+\gamma_{N}-\sigma_{N}-\epsilon_{N}}\}/\\ & & \{x_{0}\left[1+\sum_{k=1}^{N-1}\prod_{i=1}^{k}\frac{\varphi_{i-1}}{\mu_{i}+\varphi_{i}+\gamma_{i}-\sigma_{i}-\epsilon_{i}}+\frac{\varphi_{N-1}}{\mu_{N}+\gamma_{N}-\sigma_{N}-\epsilon_{N}}\prod_{i=1}^{N-1}\frac{\varphi_{i-1}}{\mu_{i}+\varphi_{i}+\gamma_{i}-\sigma_{i}-\epsilon_{i}}\right]+\\ & & \sum_{k=1}^{N-1}[\left(1-\delta_{k,1}\right)\sum_{j=1}^{k-1}\left(\frac{\kappa_{j-1}+\lambda_{j-1}-\zeta_{j-1}-\theta_{j-1}}{\mu_{j}+\varphi_{j}+\gamma_{j}-\sigma_{j}-\epsilon_{j}}\prod_{l=j+1}^{k}\frac{\varphi_{l-1}}{\mu_{l}+\varphi_{l}+\gamma_{l}-\sigma_{l}-\epsilon_{l}}\right)+\\ & & \frac{\kappa_{k-1}+\lambda_{k-1}-\zeta_{k-1}-\theta_{k-1}}{\mu_{k}+\varphi_{k}+\gamma_{k}-\sigma_{k}-\epsilon_{k}}]+\frac{\varphi_{N-1}}{\mu_{N}+\gamma_{N}-\sigma_{N}-\epsilon_{N}}\left(1-\delta_{N,2}\right)\sum_{j=1}^{N-2}(\frac{\kappa_{j-1}+\lambda_{j-1}-\zeta_{j-1}-\theta_{j-1}}{\mu_{j}+\varphi_{j}+\gamma_{j}-\sigma_{j}-\epsilon_{j}}\times \\ & & \prod_{l=j+1}^{N-1}\frac{\varphi_{l-1}}{\mu_{l}+\varphi_{l}+\gamma_{l}-\sigma_{l}-\epsilon_{l}})+\frac{\varphi_{N-1}}{\mu_{N}+\gamma_{N}-\sigma_{N}-\epsilon_{N}}\frac{\kappa_{N-2}+\lambda_{N-2}-\zeta_{N-2}-\theta_{N-2}}{\mu_{N-1}+\varphi_{N-1}+\gamma_{N-1}-\sigma_{N-1}-\epsilon_{N-1}}+\\ & & \frac{\kappa_{N-1}+\lambda_{N-1}-\zeta_{N-1}-\theta_{N-1}}{\mu_{N}+\gamma_{N}-\sigma_{N}-\epsilon_{N}}\}. \end{array}$$

To the best of our knowledge, the class of distributions (21)–(23) was not discussed by other authors. The corresponding class of distributions for the case N=∞ is discussed in Appendix A. We note that the system of Equations (16)–(18) is connected to the system of equations

(24) $$x_{i}=F_{i}x_{i-1}+\alpha_{i-1},i=1,\cdots ,N,$$

where $x_{0}$ and $\alpha_{i}$ are parameters and $F_{i}$ is a function of *i* and eventually also a function of other variables and parameters. This connection can be easily verified. We just have to set

(25) $$\begin{array}{ccc} F_{i} & = & \frac{\varphi_{i-1}}{\mu_{i}+\varphi_{i}-\gamma_{i}-\sigma_{i}-\epsilon_{i}};\\ \alpha_{i-1} & = & \frac{\kappa_{i-1}+\lambda_{i-1}+\zeta_{i-1}-\theta_{i-1}}{\mu_{i}+\varphi_{i}-\gamma_{i}-\sigma_{i}-\epsilon_{i}} \end{array}$$

in Equation (17) and

(26) $$\begin{array}{ccc} F_{N} & = & \frac{\varphi_{N-1}}{\mu_{N}-\gamma_{N}-\sigma_{N}-\epsilon_{N}};\\ \alpha_{N-1} & = & \frac{\kappa_{N-1}+\lambda_{N-1}+\zeta_{N-1}-\theta_{N-1}}{\mu_{N}-\gamma_{N}-\sigma_{N}-\epsilon_{N}} \end{array}$$

in Equation (18). In addition, we stress that $x_{0}$ is a free parameter.

Equation (24) leads to a class of probability distributions as follows. From Equation (24) we obtain

(27) $$\begin{array}{c} x_{k}=x_{0}\prod_{i=1}^{k}F_{i}+\left(1-\delta_{k1}\right)\sum_{j=1}^{k-1}\left(\alpha_{j-1}\prod_{l=j+1}^{k}F_{l}\right)+\alpha_{k-1},\;\;k=1,\cdots ,N \end{array}$$

Then the amount of substance in the channel will be

(28) $$\begin{array}{c} x=x_{0}+\sum_{k=1}^{N}x_{k}=x_{0}\left[1+\sum_{k=1}^{N}\prod_{i=1}^{k}F_{i}\right]+\sum_{k=1}^{N}\left[\left(1-\delta_{k1}\right)\sum_{j=1}^{k-1}\left(\alpha_{j-1}\prod_{l=j+1}^{k}F_{l}\right)+\alpha_{k-1}\right] \end{array}$$

We can consider probability distribution $y_{i}=x_{i}/x$ connected to the amount of substance in the nodes of the studied channel. $y_{i}$ can be considered as probability values of a distribution of a discrete random variable ω: $y_{i}=p\left(\omega =i\right)$, i=0,⋯,N. For this distribution, we obtain

(29) $$\begin{array}{ccc} y_{0} & = & \frac{x_{0}}{x_{0}\left[1+\sum_{k=1}^{N}\prod_{i=1}^{k}F_{i}\right]+\sum_{k=1}^{N}\left[\left(1-\delta_{k1}\right)\sum_{j=1}^{k-1}\left(\alpha_{j-1}\prod_{l=j+1}^{k}F_{l}\right)+\alpha_{k-1}\right]},\\ y_{k} & = & \frac{x_{0}\prod_{i=1}^{k}F_{i}+\left(1-\delta_{k1}\right)\sum_{j=1}^{k-1}\left(\alpha_{j-1}\prod_{l=j+1}^{k}F_{l}\right)+\alpha_{k-1}}{x_{0}\left[1+\sum_{k=1}^{N}\prod_{i=1}^{k}F_{i}\right]+\sum_{k=1}^{N}\left[\left(1-\delta_{k1}\right)\sum_{j=1}^{k-1}\left(\alpha_{j-1}\prod_{l=j+1}^{k}F_{l}\right)+\alpha_{k-1}\right]},\;k=1,\cdots ,N. \end{array}$$

**Figure 3:**
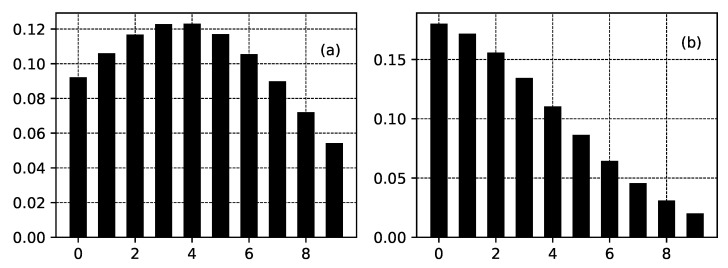

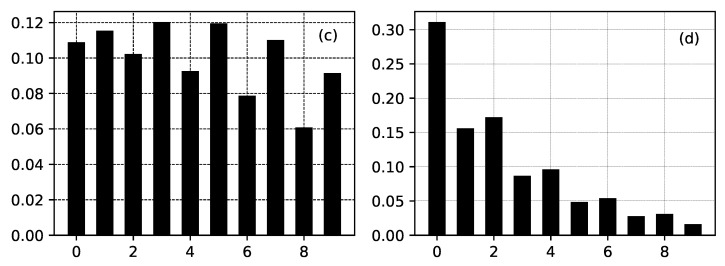
Several truncated distributions for a channel of 10 nodes connected to relationship (24) for the case of a fixed value of $\alpha_{i-1}$ and different values of $F_{i}$. $\alpha_{i-1}$ has fixed value $\alpha_{i-1}=0.2$, i=1,⋯,10 for all figures. In addition, $x_{0}=10$ for all figures. (**a**) $F_{i}=1.2-0.05i$. (**b**) $F_{i}=\exp\left(-0.05i\right)$. (**c**) $F_{i}=1-\sin\left(3.2i\right)$. (**d**) $F_{i}=0.8+0.3{\left(-1\right)}^{i}$.

**Figure 4:**
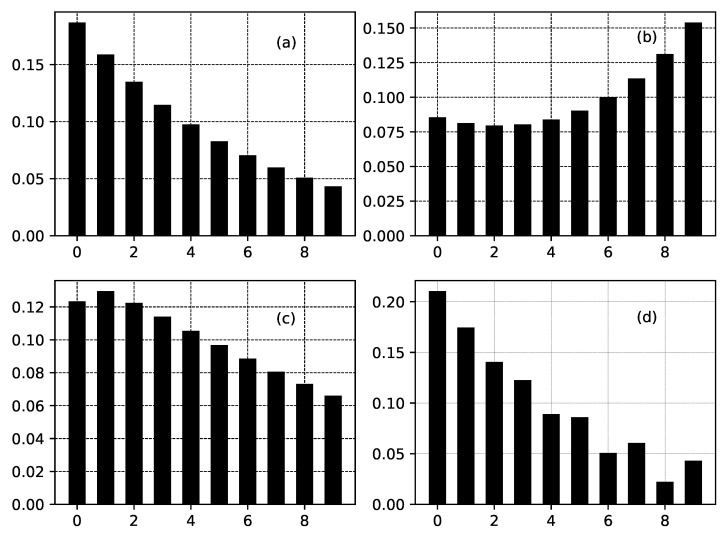
Several truncated distributions for a channel containing 10 nodes connected to relationship (24) for the case of a fixed value of $F_{i}$ and different values of $\alpha_{i-1}$. $F_{i}$ has fixed value $F_{i}=0.85$, i=1,⋯,10 for all figures. In addition $x_{0}=10$ for all figures. (**a**) $\alpha_{i}=0$. (**b**) $\alpha_{i}=\exp\left(0.2i\right)$. (**c**) $\alpha_{i}=2-i^{0.25}$. (**d**) $\alpha_{i}=-0.2+\sin\left(3.2i\right)$.

As a result of the presence of functions $F_{k}$, the shapes of the distributions from the class (29) (and the shapes of distributions from class (21), respectively) can be quite different from one another— Figure 3 and Figure 4.

Let us consider Equation (24). We note that we consider relationships connected to probability distributions and our interest will be for the values of $F_{i}$ and $x_{i}$, which are nonnegative real numbers. We shall prove the following lemma.

**Lemma** **1.** *Both quantities $F_{i}$ and $\alpha_{i}$ form Equation (24) (given by Equations (25) and (26)) can have arbitrary values at the same time.*

**Proof.**  Let us show first that $F_{i}$ can have an arbitrary (appropriate positive) real value. Indeed, in Equations (25) and (26), we have many parameters and we can choose one of them in such a way that $F_{i}$ has an appropriate arbitrary value. We obtain from Equation (25).

(30) $$\gamma_{i}=\mu_{i}+\varphi_{i}-\sigma_{i}-\epsilon_{i}-\frac{\varphi_{i-1}}{F_{i}}$$

and we can choose $\gamma_{i}$ in such a way that $F_{i}$ will be an arbitrary nonzero real number (we have just to put the desired value of $F_{i}$ in Equation (30) and we obtain the corresponding value of $\gamma_{i}$ for any combination of the other parameters and for $\varphi_{i-1}\neq 0$). There is no problem to obtain also $F_{i}=0$. For this, we just have to set $\varphi_{i-1}=0$ in Equation (25). Thus, $F_{i}$, i=1,⋯,N−1 can have arbitrary value. The same conclusion holds for $F_{N}$ and it can be obtained on the basis of Equation (26).

Let us show that the value of $\alpha_{i-1}$ in Equation (25) and the value of $\alpha_{N-1}$ in Equation (26) can be arbitrary for any value of $F_{i}$. From Equation (25), we obtain

(31) $$\theta_{i-1}=\kappa_{i-1}+\lambda_{i-1}+\zeta_{i-1}-\alpha_{i-1}\left(\mu_{i}+\varphi_{i}-\gamma_{i}-\sigma_{i}-\epsilon_{i}\right)$$

From Equation (31), we obtain the needed value of $\theta_{i-1}$ for desired arbitrary value of $\alpha_{i-1}$ and fixed values of the other parameters. Note that we can obtain an arbitrary value of $\alpha_{i-1}$ by the use of $\theta_{i-1}$ and we can do this for an arbitrary value of parameter $\gamma_{i}$.

It follows from the above that $F_{i}$ and $\alpha_{i-1}$ can have arbitrary values at the same time. □

Lemma 1 has interesting consequences. Let us prove:

**Lemma** **2.** *The relationship $x_{i}=F_{i}x_{i-1}+\alpha_{i-1}$ from Equation (24) connected to distribution (21)–(23) describes any sequence $\left\{X_{i}\right\}$ of nonnegative real numbers.*

**Proof.**  Let $\left\{X_{i}\right\}$ be an arbitrary sequence of nonnegative real numbers. We note that $x_{0}$ connected to distribution (21)–(23) can have arbitrary (nonnegative real) value. Let us set $x_{0}=X_{0}$. According to Lemma 1, $F_{1}$ and $\alpha_{0}$ can have arbitrary values at the same time. Let us consider the following equation for $X_{1}$: $X_{1}=F_{1}X_{0}+\alpha_{0}$. As $F_{1}$ and $\alpha_{0}$ can have arbitrary values, we can choose these two parameters in such a way that the equation for $X_{1}$ is satisfied for an arbitrary value of $X_{1}$ given the value of $X_{0}$, e.g., $\alpha_{0}=X_{1}-F_{1}X_{0}$. We can do the same for $X_{2}$. We can write the equation $X_{2}=F_{2}X_{2}+\alpha_{1}$ and we can choose free parameters $F_{2}$ and $\alpha_{1}$ in such a way that the equation is satisfied for arbitrary $X_{2}$ given the value of $X_{1}$. We can proceed in this way with respect to $X_{3}$, ⋯ and as we have enough free parameters, we can choose them in such a way that we can obtain any sequence of numbers $\left\{X_{i}\right\}$. Thus, the relationship $x_{i}=F_{i}x_{i-1}+\alpha_{i-1}$ from Equation (24) connected to distribution (21)–(23) describes any sequence $\left\{X_{i}\right\}$ of nonnegative real numbers. □

Lemma 2 leads us to the main result:

**Theorem** **1.** *Any truncated discrete probability distribution of the random variable ω that can take values 0,1,⋯,N is a particular case of the distribution (21)–(23).*

**Proof.**  Any discrete probability distribution $\left\{Z_{i}\right\}$, i=0,⋯,N of the random variable ω can be written in the from

(32) $$Z_{i}=F_{i}Z_{i-1}+\beta_{i-1},\;\;\;i=1,\cdots ,N$$

where $F_{i}$ has a nonnegative real value and $\beta_{i-1}$ has a real value. If the above is not true then there exists a sequence $\left\{Z_{i}^{*}\right\}$, i=0,⋯,N such that it can not be represented in the form (32). We can take $Z_{0}=Z_{0}^{*}$ and then we can construct all of the other $Z_{i}^{*}$ by relationship of the kind (32) fixing the parameters $F_{i}$ and $\beta_{i-1}$ (as it has been done in the Proof of Lemma 2). Thus, we can represent any truncated discrete probability distribution by a sequence of the kind (32). However, the sequence of kind (32) is a particular case of the sequence $x_{i}=F_{i}x_{i-1}+\alpha_{i-1}$ from Lemma 2 for the case when *x* varies between 0 and 1. Thus, any truncated discrete probability distribution can be described by a sequence of the kind $x_{i}=F_{i}x_{i-1}+\alpha_{i-1}$ from Lemma 2. The sequence $x_{i}=F_{i}x_{i-1}+\alpha_{i-1}$ from Lemma 2 describes probability distributions from the kind (21)–(23). Then the class of probability distributions described by (32) belongs to the class of probability distributions described by (21)–(23). This means that any truncated discrete probability distribution of the random variable ω is a particular case of the distribution (21)–(23). □

## 5. Discussion

In this text, we propose a discrete model of motion of substance in a channel of a network which accounts for: (i) exchanges of substance between the nodes of the channel and the nodes of the network, (ii) exchanges of substance between the nodes of the channel and the environment of the network, (iii) exchanges of substance between the flows in the channel and the nodes of the network, and (iv) exchanges of substance between the flows in the channel and the environment of the network. The general model is complicated but nevertheless one can obtain analytical results for the distribution of substance in the nodes of the channel for the particular case of stationary flow of substance through the channel. We obtain the analytical relationship for the general distribution of substance under these conditions of flow for the case of a channel containing a finite number of nodes (in Appendix A we present the corresponding distribution for the case of a channel containing an infinite number of nodes and in Appendix B we discuss the continuous version of the distribution). We show that the obtained distribution contains as particular cases all possible discrete distributions of a random variable which can take values 0,1,⋯,N. This class of distributions contains famous named distributions and entire families of discrete distributions such as families of distributions of Katz, Ord, Kemp, etc. [44,45,46,47,48,49,50,51,52,53,54].

The new point in this study is connected with the presence of the processes (5)–(8) described in Section 2. The processes are connected to exchange of substance among flows between the nodes and the environment of the studied channel. If the values of the parameters listed in points 5–12 of Section 2 are set to 0 then the model is reduced to the model discussed in [14]. Thus, the results obtained in [14] are a particular case of the results obtained in this text. In [17], we discuss a continuous model for flow of substance in a channel of a network. The probability distributions for the case of stationary flow of substance along the channel obtained in [17] are particular cases of the corresponding probability distributions obtained in this text and this is a consequence of Theorem 2 from Appendix A.

The theory discussed above leads to interesting results and can have numerous applications. We have started our research on flows in channels of the network by a study of problems of migration [27,28,29,30,31]. The theory presented above can be applied for many other practical situations connected to the flow of substances in networks. The flows can be, for example, traffic flows and the substances can be resources, goods, or even humans. The discussed model has potential to describe various situations connected to different kinds of flows and one can obtain analytical results for cases of simple flows or can make numerical simulations for the case of more complicated flows.

## Figures and Tables

**Figure A1:**
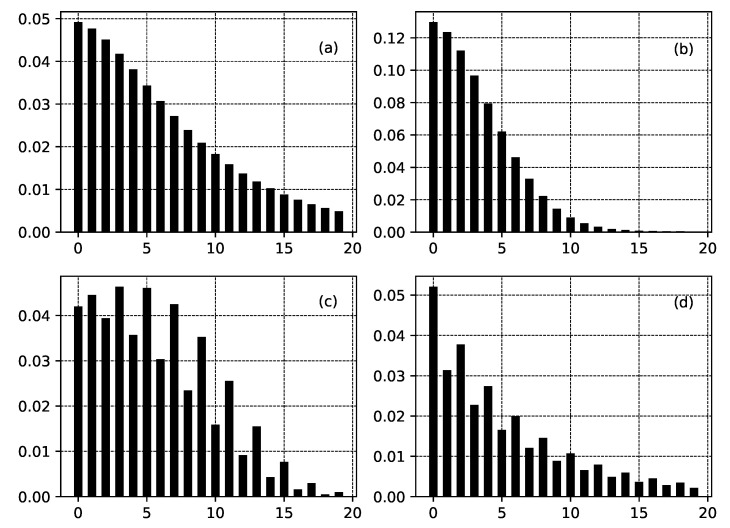
Several distributions connected to the relationship (A8) for the case of fixed value of $\alpha_{i-1}$ and different values of $F_{i}$. $\alpha_{i-1}$ has the fixed value $\alpha_{i-1}=0.2$, i=1,⋯ for all figures. In addition, $x_{0}=10$ for all figures. (**a**) $F_{i}=1.01-0.2i/\left(i+4\right)$. (**b**) $F_{i}=\exp\left(-0.05i\right)$. (**c**) $F_{i}=1-\sin\left(3.2i\right)$. (**d**) $F_{i}=0.9+0.3{\left(-1\right)}^{i}$.

**Figure A2:**
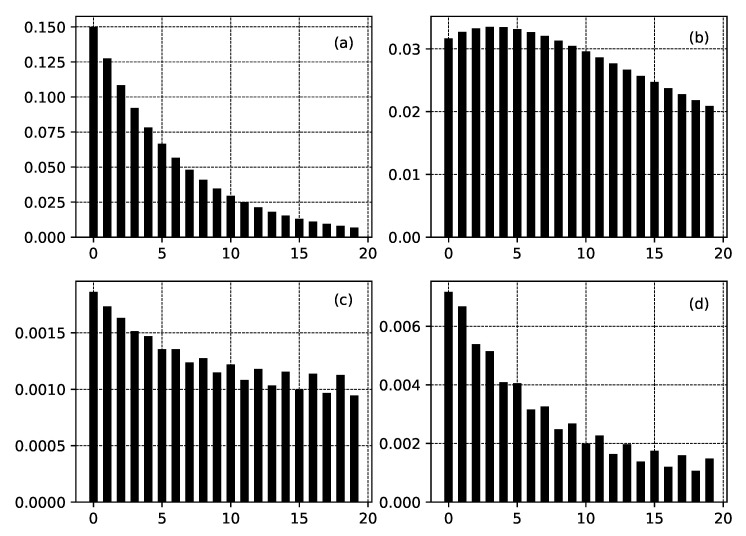
Several distributions connected to the relationship (A8) for the case of a fixed value of $F_{i}$ and different values of $\alpha_{i-1}$. $F_{i}$ has the fixed value $F_{i}=0.85$, i=1,⋯ for all figures. In addition $x_{0}=10$ for all figures. (**a**) $\alpha_{i}=0$. (**b**) $\alpha_{i}=\exp\left(0.6-0.05i\right)$. (**c**) $\alpha_{i}=0.8-\sin3.2i$. (**d**) $\alpha_{i}=0.2+0.6{\left(-1\right)}^{i}$.

**Figure A3:**
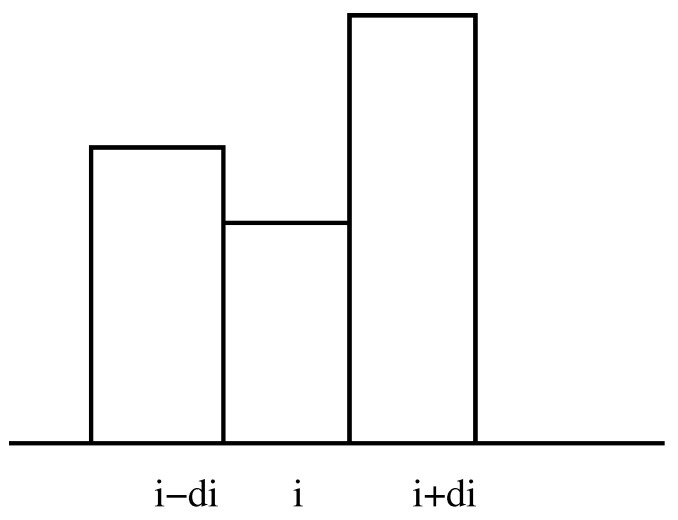
Values of x(i) for the intervals i−di, *i*, and i+di.

## Appendix A. Probability Distributions of Substance for a Channel Containing an Infinite Number of Nodes

The model of a channel containing an infinite number of nodes corresponding to the model of a channel containing a finite number of nodes and described by Equations (13)–(15) is as follows

(A1) $$\begin{array}{c} \left[\sigma_{0}\left(t_{k}\right)-\mu_{0}\left(t_{k}\right)-\varphi_{0}\left(t_{k}\right)-\gamma_{0}\left(t_{k}\right)+\epsilon_{0}\left(t_{k}\right)\right]x_{0}\left(t_{k}\right)=0, \end{array}$$

(A2) $$\begin{array}{c} \left[\mu_{i}\left(t_{k}\right)+\varphi_{i}\left(t_{k}\right)+\gamma_{i}\left(t_{k}\right)-\sigma_{i}\left(t_{k}\right)-\epsilon_{i}\left(t_{k}\right)\right]x_{i}\left(t_{k}\right)=\varphi_{i-1}\left(t_{k}\right)x_{i-1}\left(t_{k}\right)+(-\zeta_{i-1}\left(t_{k}\right)-\theta_{i-1}\left(t_{k}\right)+\\ \kappa_{i-1}\left(t_{k}\right)+\lambda_{i-1}\left(t_{k}\right)),\;\;i=1,\cdots \end{array}$$

We consider the case when the parameters of the model are time independent (i.e., when $\sigma_{i}\left(t_{k}\right)=\sigma$; $\mu_{i}\left(t_{k}\right)=\alpha_{i}$; $\gamma_{i}\left(t_{k}\right)=\gamma_{i}$; $\epsilon_{i}\left(t_{k}\right)=\epsilon_{i}$; $\varphi_{0}\left(t_{k}\right)=\varphi_{0}$; i=0,⋯). In this case, the system of equations becomes

(A3) $$\begin{array}{c} \left[\sigma_{0}-\mu_{0}-\varphi_{0}-\gamma_{0}+\epsilon_{0}\right]x_{0}=0 \end{array}$$

(A4) $$\begin{array}{c} \left[\mu_{i}+\varphi_{i}+\gamma_{i}-\sigma_{i}-\epsilon_{i}\right]x_{i}=\varphi_{i-1}x_{i-1}+\left(-\zeta_{i-1}-\theta_{i-1}+\kappa_{i-1}+\lambda_{i-1}\right),\;\;i=1,\cdots \end{array}$$

From Equations (A3) and (A4), we obtain the relationships

(A5) $$\begin{array}{ccc} \varphi_{0} & = & \sigma_{0}-\mu_{0}-\gamma_{0}+\epsilon_{0};\\ x_{k} & = & x_{0}\prod_{i=1}^{k}\frac{\varphi_{i-1}}{\mu_{i}+\varphi_{i}+\gamma_{i}-\sigma_{i}-\epsilon_{i}}+\left(1-\delta_{k1}\right)\sum_{j=1}^{k-1}\left(\frac{\kappa_{j-1}+\lambda_{j-1}-\zeta_{j-1}-\theta_{j-1}}{\mu_{j}+\varphi_{j}+\gamma_{j}-\sigma_{j}-\epsilon_{j}}\prod_{l=j+1}^{k}\frac{\varphi_{l-1}}{\mu_{l}+\varphi_{l}+\gamma_{l}-\sigma_{l}-\epsilon_{l}}\right)+\\ & & \frac{\kappa_{k-1}+\lambda_{k-1}-\zeta_{k-1}-\theta_{k-1}}{\mu_{k}+\varphi_{k}+\gamma_{k}-\sigma_{k}-\epsilon_{k}},\;k=1,\cdots \end{array}$$

From Equation (A5), we obtain

(A6) $$\begin{array}{ccc} x & = & \sum_{k=0}^{\infty }x_{k}=x_{0}\left[1+\sum_{k=1}^{\infty }\prod_{i=1}^{k}\frac{\varphi_{i-1}}{\mu_{i}+\varphi_{i}+\gamma_{i}-\sigma_{i}-\epsilon_{i}}\right]+\\ & & \sum_{k=1}^{\infty }\left[\left(1-\delta_{k,1}\right)\sum_{j=1}^{k-1}\left(\frac{\kappa_{j-1}+\lambda_{j-1}-\zeta_{j-1}-\theta_{j-1}}{\mu_{j}+\varphi_{j}+\gamma_{j}-\sigma_{j}-\epsilon_{j}}\prod_{l=j+1}^{k}\frac{\varphi_{l-1}}{\mu_{l}+\varphi_{l}+\gamma_{l}-\sigma_{l}-\epsilon_{l}}\right)+\frac{\kappa_{k-1}+\lambda_{k-1}-\zeta_{k-1}-\theta_{k-1}}{\mu_{k}+\varphi_{k}+\gamma_{k}-\sigma_{k}-\epsilon_{k}}\right] \end{array}$$

We consider probability distribution $y_{i}=x_{i}/x$ for the amount of substance in the nodes of the studied channel. $y_{i}$ can be considered as probability values of the distribution of a discrete random variable ω: $y_{i}=p\left(\omega =i\right)$, i=1,⋯,∞. For this distribution, we obtain

(A7) $$\begin{array}{ccc} y_{0} & = & x_{0}/\{x_{0}\left[1+\sum_{k=1}^{\infty }\prod_{i=1}^{k}\frac{\varphi_{i-1}}{\mu_{i}+\varphi_{i}+\gamma_{i}-\sigma_{i}-\epsilon_{i}}\right]+\\ & & \sum_{k=1}^{\infty }\left[\left(1-\delta_{k,1}\right)\sum_{j=1}^{k-1}\left(\frac{\kappa_{j-1}+\lambda_{j-1}-\zeta_{j-1}-\theta_{j-1}}{\mu_{j}+\varphi_{j}+\gamma_{j}-\sigma_{j}-\epsilon_{j}}\prod_{l=j+1}^{k}\frac{\varphi_{l-1}}{\mu_{l}+\varphi_{l}+\gamma_{l}-\sigma_{l}-\epsilon_{l}}\right)+\frac{\kappa_{k-1}+\lambda_{k-1}-\zeta_{k-1}-\theta_{k-1}}{\mu_{k}+\varphi_{k}+\gamma_{k}-\sigma_{k}-\epsilon_{k}}\right]\},\\ y_{k} & = & \{x_{0}\prod_{i=1}^{k}\frac{\varphi_{i-1}}{\mu_{i}+\varphi_{i}+\gamma_{i}-\sigma_{i}-\epsilon_{i}}+\left(1-\delta_{k1}\right)\sum_{j=1}^{k-1}\left(\frac{\kappa_{j-1}+\lambda_{j-1}-\zeta_{j-1}-\theta_{j-1}}{\mu_{j}+\varphi_{j}+\gamma_{j}-\sigma_{j}-\epsilon_{j}}\prod_{l=j+1}^{k}\frac{\varphi_{l-1}}{\mu_{l}+\varphi_{l}+\gamma_{l}-\sigma_{l}-\epsilon_{l}}\right)+\\ & & \frac{\kappa_{k-1}+\lambda_{k-1}-\zeta_{k-1}-\theta_{k-1}}{\mu_{k}+\varphi_{k}+\gamma_{k}-\sigma_{k}-\epsilon_{k}}\}/\{x_{0}\left[1+\sum_{k=1}^{\infty }\prod_{i=1}^{k}\frac{\varphi_{i-1}}{\mu_{i}+\varphi_{i}+\gamma_{i}-\sigma_{i}-\epsilon_{i}}\right]+\\ & & \sum_{k=1}^{\infty }[\left(1-\delta_{k,1}\right)\sum_{j=1}^{k-1}\left(\frac{\kappa_{j-1}+\lambda_{j-1}-\zeta_{j-1}-\theta_{j-1}}{\mu_{j}+\varphi_{j}+\gamma_{j}-\sigma_{j}-\epsilon_{j}}\prod_{l=j+1}^{k}\frac{\varphi_{l-1}}{\mu_{l}+\varphi_{l}+\gamma_{l}-\sigma_{l}-\epsilon_{l}}\right)+\\ & & \frac{\kappa_{k-1}+\lambda_{k-1}-\zeta_{k-1}-\theta_{k-1}}{\mu_{k}+\varphi_{k}+\gamma_{k}-\sigma_{k}-\epsilon_{k}}]\}\;\;k=1,\cdots ,\infty \end{array}$$

To the best of our knowledge, the class of distributions (A7) was not discussed by other authors.

We note that the system of Equations (A3) and (A4) is connected to the system of equations

(A8) $$x_{i}=F_{i}x_{i-1}+\alpha_{i-1},i=1,\cdots ,$$

where $x_{0}$ and $\alpha_{i}$ are parameters and $F_{i}$ is a function of *i* and eventually also a function of other variables and parameters. This connection can be easily proved. We have just to set

(A9) $$\begin{array}{c} F_{i}=\frac{\varphi_{i-1}}{\mu_{i}+\varphi_{i}-\gamma_{i}-\sigma_{i}-\epsilon_{i}};\;\;\;\alpha_{i-1}=\frac{\kappa_{i-1}+\lambda_{i-1}+\zeta_{i-1}-\theta_{i-1}}{\mu_{i}+\varphi_{i}-\gamma_{i}-\sigma_{i}-\epsilon_{i}} \end{array}$$

in Equation (A4). In addition, we stress that $x_{0}$ is a free parameter.

Equation (A8) leads to a class of probability distributions as follows. From Equation (A8), we obtain

(A10) $$\begin{array}{c} x_{k}=x_{0}\prod_{i=1}^{k}F_{i}+\left(1-\delta_{k1}\right)\sum_{j=1}^{k-1}\left(\alpha_{j-1}\prod_{l=j+1}^{k}F_{l}\right)+\alpha_{k-1},\;\;\;k=1,\cdots \end{array}$$

Then the amount of substance in the studied channel is

(A11) $$\begin{array}{c} x=x_{0}+\sum_{k=1}^{\infty }x_{k}=x_{0}\left[1+\sum_{k=1}^{\infty }\prod_{i=1}^{k}F_{i}\right]+\sum_{k=1}^{\infty }\left[\left(1-\delta_{k1}\right)\sum_{j=1}^{k-1}\left(\alpha_{j-1}\prod_{l=j+1}^{k}F_{l}\right)+\alpha_{k-1}\right] \end{array}$$

We consider probability distribution $y_{i}=x_{i}/x$ for the amount of substance in the nodes of the channel. $y_{i}$ can be considered as probability values of distribution of a discrete random variable ω: $y_{i}=p\left(\omega =i\right)$, i=0,⋯. For this distribution, we obtain

(A12) $$\begin{array}{ccc} y_{0} & = & \frac{x_{0}}{x_{0}\left[1+\sum_{k=1}^{\infty }\prod_{i=1}^{k}F_{i}\right]+\sum_{k=1}^{\infty }\left[\left(1-\delta_{k1}\right)\sum_{j=1}^{k-1}\left(\alpha_{j-1}\prod_{l=j+1}^{k}F_{l}\right)+\alpha_{k-1}\right]};\\ y_{k} & = & \frac{x_{0}\prod_{i=1}^{k}F_{i}+\left(1-\delta_{k1}\right)\sum_{j=1}^{k-1}\left(\alpha_{j-1}\prod_{l=j+1}^{k}F_{l}\right)+\alpha_{k-1}}{x_{0}\left[1+\sum_{k=1}^{\infty }\prod_{i=1}^{k}F_{i}\right]+\sum_{k=1}^{\infty }\left[\left(1-\delta_{k1}\right)\sum_{j=1}^{k-1}\left(\alpha_{j-1}\prod_{l=j+1}^{k}F_{l}\right)+\alpha_{k-1}\right]},\;\;k=1,\cdots \end{array}$$

Several probability distributions connected to relationships (A8) and (A12) are shown in Figure A1 and Figure A2.

Let us consider (A8). We note that we consider relationships connected to probability distributions and our interest will be for values of $F_{i}$ and $x_{i}$ that are nonnegative real numbers. Analogous to the proofs in the main text, we can prove two lemmas and a theorem as follows.

**Lemma** **A1.**
*Both quantities $F_{i}$ and $\alpha_{i}$ form (A8) (given by (A9)) can have arbitrary values at the same time.*

**Lemma** **A2.**
*The relationship $x_{i}=F_{i}x_{i-1}+\alpha_{i-1}$ from (A8) connected to distribution (A7) describes any sequence $\left\{X_{i}\right\}$ of nonnegative real numbers.*

**Theorem** **A1.**
*Any discrete probability distribution of the random variable ω, which can take values 0,1,⋯, is a particular case of distribution (A7).*

For the case of a continuous distribution, we can construct a discrete distribution that is a discrete analogue of the continuous distribution. Let for an example *X* be a continuous variable (x∈[0,∞)) with p.d.f. f(x). The discrete analogue of f(x) can be obtained as follows: one considers discrete random variable *Y* with possible values Y=k, k=0,1,⋯, and p.m.f.

(A13) $$P\left(Y=k\right)=\frac{f(k)}{\sum_{l=0}^{\infty }f\left(l\right)},\;\;\;k=0,1,\cdots ,\;\;l=0,1,\cdots$$

Then we can formulate:

**Theorem** **A2.**
*Any discrete analogue of the kind (A13) of a continuous probability distribution is a particular case of the distribution (A7).*

**Proof.** Any distribution of kind (A13) is a discrete probability distribution of a random variable of the random variable ω from the Theorem 2. Then, according to Theorem 2, this distribution is a particular case of the distribution (A7). □

There exists also other ways for construction of discrete analogues of continuous distributions (see, e.g., [55,56]). Let us consider the discrete analogues that are discrete distributions of a discrete random variable *Y* with possible values Y=k, k=0,1,⋯. Let us denote as C the class of these discrete distributions. Then we can extend Theorem 3 to

**Theorem** **A3.**
*Any discrete analogue of a continuous probability distribution that is a member of the class C is a particular case of the distribution (A7).*

**Proof.** Any distribution from the class C is a discrete probability distribution of a random variable of the kind of the random variable ω from the Theorem 2. Then, according to Theorem 2, this distribution is a particular case of the distribution (A7). □

## Appendix B. A General Continuous Distribution

Below, we apply an approach analogous to the approach in the main text in order to obtain a continuous probability distribution containing known continuum distributions as particular cases. We consider a function x(i) where *i* is a continuous real variable which takes values in the interval from $i_{1}$ to $i_{2}$ (e.g., $i_{1}$ can be −∞ and $i_{2}$ can be +∞ or $i_{1}$ can be 0 and $i_{2}$ can be +∞). The interval for *i* is divided into intervals of infinitesimal length di and we assume that the values of x(i) are nonnegative real values.

Figure A3 shows one example of possible values of x(i) for the values i−di, *i*, and i+di of quantity *i*. We remember that for the case of discrete values of *i* in the main text (see Equation (24)), we have $x_{i}=F_{i}x_{i-1}+\alpha_{i-1}$, i=1,⋯,N. For the case of continuous values of *i*, we shall start from relationship

(A14) $$\begin{array}{ccc} \frac{dx}{di} & = & F(i)x(i)+\alpha (i) \end{array}$$

In (A14), F(i) and α(i) can be arbitrary real functions of *i*. The only condition is that x(i) has to be nonnegative. In such a way, (A14) can describe arbitrary shape of the distribution x(i).

The solution of (A14) is as follows

(A15) $$\begin{array}{c} x\left(i\right)=\exp\left[\int di\;F(i)\right]\{C+\\ \int di\left[\alpha \left(i\right)\exp\left(-\int di\;F(i)\right)\right]\} \end{array}$$

where *C* is a constant of integration.

For the case when *i* can have values in the interval from $i_{1}$ and $i_{2}$, we have

$$\begin{array}{c} x^{*}=\int_{i_{1}}^{i_{2}}di\{\exp\left[\int di\;F(i)\right]\{C+\\ \int di\left[\alpha \left(i\right)\exp\left(-\int di\;F(i)\right)\right]\}\} \end{array}$$

and then we can construct the probability distribution

(A16) $$\begin{array}{c} y\left(i\right)=\frac{x(i)}{x^{*}}=\\ \frac{\exp\left[\int di\;F(i)\right]\left\{C+\int di\left[\alpha \left(i\right)\exp\left(-\int di\;F(i)\right)\right]\right\}}{\int_{i_{1}}^{i_{2}}di\left\{\exp\left[\int di\;F(i)\right]\left\{C+\int di\left[\alpha \left(i\right)\exp\left(-\int di\;F(i)\right)\right]\right\}\right\}} \end{array}$$

The distribution (A16) contains two arbitrary functions: F(i) and α(i) and because this is a very general one, in other words, it contains as particular cases all continuous statistical distributions which satisfy Equation (A14). Let us show now that any distribution D(i) where *i* takes values from $i_{1}$ to $i_{2}$ is a particular case of the distribution (A16). In order to show this, we have to satisfy the relationship

(A17) $$\begin{array}{c} D\left(i\right)=\exp\left[\int di\;F(i)\right]\{C+\\ \int di\left[\alpha \left(i\right)\exp\left(-\int di\;F(i)\right)\right]\} \end{array}$$

Then,

$$\begin{array}{c} \int_{i_{1}}^{i_{2}}di\;D\left(i\right)=\int_{i_{1}}^{i_{2}}di\exp\left[\int di\;F(i)\right]\{C+\\ \int di\left[\alpha \left(i\right)\exp\left(-\int di\;F(i)\right)\right]\}=1 \end{array}$$

We shall satisfy the relationship (A17), for example, by choosing an appropriate form of the arbitrary function α(i) (we can fix also F(i)). For the required from of α(i), we obtain from (A17)

(A18) $$\begin{array}{ccc} \alpha (i) & = & \exp\left[\int diF(i)\right]\frac{d}{di}\left[D\left(i\right)\exp\left(-\int diF(i)\right)\right] \end{array}$$

For an example, if we want D(i) to be the normal distribution

(A19) $$D\left(i\right)=\frac{1}{{\left(2\pi \sigma \right)}^{1/2}}\exp\left(-\frac{{\left(i-\mu \right)}^{2}}{2\sigma^{2}}\right)$$

we have to set for α(i)

(A20) $$\begin{array}{c} \alpha \left(i\right)=\frac{1}{{\left(2\pi \sigma \right)}^{1/2}}\exp\left[\int diF(i)\right]\times \\ \frac{d}{di}\left[\exp\left(-\frac{{\left(i-\mu \right)}^{2}}{2\sigma^{2}}\right)\exp\left(-\int diF(i)\right)\right] \end{array}$$
